# Supplementary material for: Development and validation of audio-based guided imagery and progressive muscle relaxation tools for functional bloating
Source: PLoS One. 2022 Sep 26;17(9):e0268491. doi: 10.1371/journal.pone.0268491 (PMC9512190; doi:10.1371/journal.pone.0268491)
Supplement: S1 File — (DOCX) [file pone.0268491.s001.docx]

**Supporting Information**

**Supporting Information 1**

**Interview Guide (Pre)**

1. Clinical history that focuses on symptoms and precipitating factors

- Symptoms: pattern, severity, frequency, duration of illness
- Possible precipitating factors prior to onset: GI infection, lifestyle changes
- Impact of symptoms: psychological effects, avoidance, relationships, work
- Coping strategies: what is or not helpful?
- General lifestyle: eating habits, work, stress

2. Psychosocial and Personality factors

- How would you describe your personality; how others describe you? Get stressed easily?
- Major life events and ongoing minor hassles
- Relationship difficulties
- Hectic schedule (skipping meals)
- Irrational beliefs, high expectations (should, must)
- Feeling guilty about attending to own needs
- Need for social approval, pleasing others
- Low self-esteem and confidence
- Poor coping styles (what they do when they are stressed)
- External locus of control; low self-efficacy, helplessness
- Cognitive distortions (catastrophizing, generalizing)
- Overresponsibility for others

**Supporting Information 2**

Lists of songs included in the ratings

| Song | Title | Author | Descriptions |
| --- | --- | --- | --- |
| Song 1 | Bamboo Flute | Chen Yue | Traditional Chinese |
| Song 2 | The Old Tree | Peder B. Helland | Classical Harp |
| Song 3 | River of Sounds | Jutesets | Jazz |
| Song 4 | Uplifting Hope | Grey Houston | Alpha Waves |
| Song 5 | Moonlight Sonata | Beethoven | Classical Music |
| Song 6 | Deep Sleep in 10 minutes | Aishwarya Tripathi | Sivananda Yoga |
| Song 7 | Delta Wave Binaural Beats | Zac Kaiser | 3.2Hz Delta Brainwaves |
| Song 8 | Javanese Cinematic Backsound | Joe Karnan | Indonesian Instrumental |
| Song 9 | 10 minute Freestyle Beats | prod. 45LIMIT | Rap Instrumental Beat |
| Song 10 | Behind Barz | Anabolic Beatz | Boom Bap Rap Beat |
| Song 11 | Laid-back Acoustic Blues Guitar | Justin Johnson | Blues Guitar |
| Song 12 | Rock Hip-Hop Instrumental Beat | Makeeng Prod | Rock and Roll Beat |
| Song 13 | The GURU is inside | Kamal Sabran | Traditional Malay Lo-fi |

**Supporting Information 3**

**Interview Guide (Post)**

What came into your mind when we tell you about a relaxation audio that could help relief your bloating symptoms?

Was the technique helpful? If yes, how? If no, why?

How did you find the language, speed, instructions, dialect, background music?

What difficulties did you face during the practice? (disturbance? Understanding instructions/language?)

What effects did you experience during the practice? (flatulence, burp, pain, nausea, vomiting)

How do you feel after the practice?

How do you feel about the app? (functionality, designs, easy to use?)

Any recommendations? Suggestions to improve?

**Supporting Information 4**

| Name | Frequency | Activity |
| --- | --- | --- |
| **δ – delta** | Less than 4 Hz | Slowest and strongest brainwave, very low frequency with high amplitude. Usually associated with deep sleep or dreamless sleep. Also found during deep meditation. |
| **θ – theta** | 4 – 8 Hz | Drowsiness, light sleep, deep relaxation, or meditation. |
| **α – alpha** | 8 -12 Hz | Relaxation, calmness, or peaceful state |
| **β – beta** | 12 -30 Hz | Normal waking consciousness, high alert, active thinking, anxious, or focus. Most people operate in this band durig the day. |

Adapted from (cite Surangsrirat et al., 2015 and Zhao et al., 2018)

**Supporting Information 5**

Results of Content Validaton and Face Validation of the 2 Audios
