# Supplementary material for: Development and validation of audio-based guided imagery and progressive muscle relaxation tools for functional bloating
Source: PLoS One. 2022 Sep 26;17(9):e0268491. doi: 10.1371/journal.pone.0268491 (PMC9512190; doi:10.1371/journal.pone.0268491)
Supplement: Attachment — Submitted filename: PLOS ONE Review D-22-12725.docx [file pone.0268491.s002.docx]

PLOS ONE Review

PONE-D-22-12725

Thank you for the opportunity to review this manuscript. The authors have done very good work designing and executing a well-constructed study to address a health concern that has a significant impact on quality of life and other dimensions of health. The authors represent an experienced and multidisciplinary team well prepared to conduct this type of study.

Overall, the authors have done excellent work in describing their study with great detail and clarity. They have been clear describing each step of the study, their process and efforts to develop the tools and resources to address the issue of bloating and the measures they implemented in the study.

On page 2 (last paragraph) the authors have cited no literature related to guided imagery. The literature cited in this paragraph is only related to progressive muscle relaxation. There is a good body of evidence surrounding the use of guided imagery and this should be integrated into the manuscript. Given the study is focused on development and validation of audio-based guided imagery, literature related to guided imagery needs to be reviewed to support the development of the guided imagery intervention. The authors indicate the scripts were developed based on literature reviews. Sources should be cited in the introduction.

On page 3, in the review of literature and theoretical frameworks section there is limited review of literature, rather it focuses only on studies that were adopted to develop content. There is no review of literature that indicates a review of existing literature that further helped inform the researchers conceptualization of this study. This review of literature is so brief, I would recommend providing more context… this may need to go in the introduction rather than in the method section.

The authors have done very good work indicating the importance of cultural considerations (language and music) and how they integrated current research with these considerations at the forefront. It is important to also draw upon literature surrounding this to cite this in the manuscript.

The authors have provided a clear description of the study methods and each of the measurement tools. The authors do not detail how the imagery themes were determined. Detail is needed for readers to understand how the authors analyzed this qualitative data from study participants.

On page 12, Line 316, the authors state that the development of imagery scripts involves a thorough literature review. This is not evident from what is represented in this manuscript. While the body of literature surrounding the use of GI may be limited for treating bloating, the authors should search related literature to support the use of GI in their study.

Addressing the issues surrounding the literature as it relates to the topic will help to strength the manuscript and support the strong claims of having conducted a thorough review of the literature.
